# Patterns of Internet and smartphone use by parents of children with chronic kidney disease

**DOI:** 10.1371/journal.pone.0212163

**Published:** 2019-02-12

**Authors:** Deise Garrido, Andreia Watanabe, Ana Lídia Ciamponi, Taciana Mara Couto, Levy Anderson César Alves, Ana Estela Haddad

**Affiliations:** 1 Department of Orthodontics and Pediatric Dentistry, School of Dentistry, University of São Paulo, São Paulo, SP, Brazil; 2 Telehealth Center, School of Dentistry, University of São Paulo, São Paulo, SP, Brazil; 3 Pediatric Nephrology, Department of Pediatrics, Children's Institute, Clinical Hospital, School of Medicine, University of São Paulo, São Paulo, SP, Brazil; University of Helsinki, FINLAND

## Abstract

**Background:**

Smartphones have become a part of universal technology by combining mobile and handheld functions, enabling expanded access to health information sources available on the Internet.

The purpose of this study was to describe the pattern of smartphones and Internet use to search for health information by parents of children with chronic kidney disease (CKD).

**Methods:**

In a cross-sectional study, a questionnaire was applied to 111 parents of patients in a Brazilian pediatric nephrology center. Descriptive assessments were performed on Internet use patterns, and associative analyses were made of the influence of the smartphone use pattern on the search for health information.

**Results:**

Of the 111 participants, 91% (101/111) accessed the Internet, 88% (89/101) searched for health information, and 90% (80/89) searched for CKD information. Smartphones were the most commonly used devices to access the Internet. There was no significant difference between age groups, schooling levels, places of residence and smartphone use to search information about CKD. Physicians continue to be primary sources of information (87%, 88/101), but now they share space with the Internet, which surpassed traditional sources such as books and other health professionals. There seems to be some discomfort on the part of the parents in admitting their research habit to the physician, considering that 65% (52/80) said they did not discuss the fact that they had looked for information on the Internet with their doctor. Obtaining more information about the disease and gaining knowledge regarding its complications were the main reasons that led to performing a search on the Internet, whose results were considered useful by 93% (74/80).

**Conclusion:**

Parents of children with CKD have been using the Internet largely through smartphones to research about CKD, irrespective of age, schooling and place of residence. Given its wide use, the Internet can be an important vehicle for health education and contribute to providing the support needed by parents and patients to cope with the disease.

## Introduction

Chronic kidney disease (CKD) is devastating in children and associated with high mortality in patients in its final stages [1]. In Brazil, epidemiological data are scarce, and the prevalence of pediatric advanced stages than final stages disease in 2012 was estimated at 20 cases per million age-related population (pmarp) [2]. Throughout the course of the disease, monitoring and treatment require that the care can be shared among health professionals and family members. Difficulties and failures in treatment management can negatively influence disease outcomes [3,4,5]. Caregiving is often complex, requiring specific skills from the family members involved, who often lack the knowledge, education and understanding needed to follow the instructions given by health professionals [6,7]. In this last respect, the Internet can provide benefits to health care, and promote the empowerment of individuals, when used as a source of health information [8]; this can be especially useful for parents of children with CKD [6,9,10]. It is estimated that 40% of the world’s population uses the Internet [11]. Access has been driven by rapid growth in the use of smartphones, with 95% of the world’s population living in areas covered by mobile phone networks [11]. The use of the Internet has become commonplace in Europe and the United States (US) [12,13] as a way of obtaining health information. In the US, 72% of adult Internet users sought health information, and half (52%) used cell phones [12]. In Europe, six out of ten individuals used the Internet in 2014 as a source of health information. The main interest was for more specific information on diseases, especially on symptoms [13]. In Brazil, 58% of the population aged 10 years and over had access to the Internet in 2015, and 41% sought health information. Mobile phones were the main Internet access device for 56% of Brazilians and represented the only access device available for the majority of the less educated individuals, for the lower social classes and for dwellers in rural areas [14]. Research shows that the frequency of parents of children with some chronic, serious or rare illness who searched on the Internet is especially high [15–20]. In 2002, Cargill and Watson [3] conducted a study among parents and patients of a nephrology clinic at a hospital in the city of Nottingham (United Kingdom) to assess the frequency of use of different sources of information on kidney disease, including the Internet, TV, videos, articles in newspapers and magazines, books, booklets and leaflets produced by the clinic itself or by third parties. Although the authors found that the Internet was not the predominant source for parents of children and adolescents with general renal or chronic renal failure (20% and 23%, respectively), they suggest that the Internet would gain importance as the number of websites on the subject increased. Since the study by Cargill and Watson [3] was published, the Internet has expanded and cell phones have become a part of genuinely universal technology [11], expanding the possibility of access to health information and health care [8,21,22]. To our knowledge, the pattern of Internet use and the influence of smartphone use to search for health and CKD information by parents of pediatric patients affected by the disease is, as yet, unknown. Our primary aim is to know if this increase in access to the Internet through smartphones has allowed information about CKD to be obtained by the parents of children and adolescents with the disease, regardless of age, schooling and place of residence. The secondary aim of this study is to describe the current trend of the Internet and smartphone use pattern for the search of health information by parents of children with CKD in Brazil.

## Methods

This investigation was conducted according to the Declaration of Helsinki. The study was approved by the research ethics committees of the Children's Institute, Clinical Hospital, School of Medicine and of the School of Dentistry, University of São Paulo (no. 1.235.212). Written informed consent was obtained from all the participants.

### Setting and participants

This was a descriptive and analytical cross-sectional study. The Children's Institute was chosen as the location for data collection, because it is a Brazilian public hospital of reference in pediatric nephrology, and receives patients from all over Brazil. Although there are specialized centers in all Brazilian regions, patients and families resort to regions with greater socioeconomic development in search of more adequate treatment [2].

Parents or caregivers of 111 patients (<18 years) with CKD, stages 1 through 5, and/or renal transplant recipients were included in the period from September to October 2015. Whenever there was more than one family member accompanying the patient, the one with the longest time of interaction with the patient was chosen. Parents who did not live with the patient, and parents of children whose diagnosis had not yet been established were not included.

### Questionnaire analysis

To date, there is no validated tool for researching the behavior of parents and/or caregivers regarding the search for health information on the Internet. A descriptive and analytical cross-sectional study was carried out using a questionnaire that was based on that of previous studies [23–25] and applied in the form of individual interviews (S1 Questionnaire). A pilot study was carried out with 10 parents to evaluate the adequacy of the construction, the clarity and understanding of the questions, and the duration of questionnaire application. Data from this pilot study were not included.

The questionnaire contained questions with sociodemographic data (age, schooling, place of residence), age of the patient, relationship with the patient and information about Internet use and search for health information. Because non-parental caregivers represented only 2% (2/111) of the sample, we used the term “parents” to designate both these and actual parents.

The questionnaire was included in the QuickTapSurvey 6.2.3 research application (www.quicktapsurvey.com; TabbleDabble Inc., Toronto, Canada) on an iPad device (Apple Inc., Cupertino, CA, USA).http://www.quicktapsurvey.com/ The interviews were conducted before routine patient consultations, by a single interviewer (D.G.), in a confidential manner.

### Statistical analysis

Descriptive analyses of the data related to the use of the Internet to search for health information were performed, and a distribution of frequencies for each item was obtained. Fisher's exact test was used to evaluate the associations between the explanatory variables—namely, place of residence, age group, schooling, and Internet access through a smartphone—and the search for health and CKD information. All analyses were performed using R software, version 3.3.1 (R Foundation) [26]. The level of significance was set at p < 0.05.

## Results

### Participants

Of the 111 participants, 90% (100/111) were mothers of the patients. The majority were in the age groups of 24–34 years (42/111, 38%) and 35–44 years (44/111, 40%). With regard to schooling, 73% (81/111) had finished high school, and 20% (22/111), had a college degree (Table 1). Almost all parents (107/111, 96%) said they knew the name of their child’s illness. The patients were between 1 and 18 years of age, with an average of 10 years (SD = 4.5 years).

**Table 1:** Sociodemographic characteristics of study participants (n = 111).

Variable		N	%
Relationship with the child	Mother	100	90.0%
	Father	9	8%
	Other caretaker	2	2%
Age group	24–34 years	42	38%
	35–44 years	44	40%
	45–59 years	25	22%
Schooling	Primary	8	7%
	High school	81	73%
	College	22	20%
Place of residence^(1)^	São Paulo (city)	74	67%
	Interior of São Paulo	17	15%
	Other Brazilian states	20	18%

Note

(1) The city of São Paulo is the capital of the Brazilian state also called São Paulo.

### General Internet use characteristics

**Fig 1:**
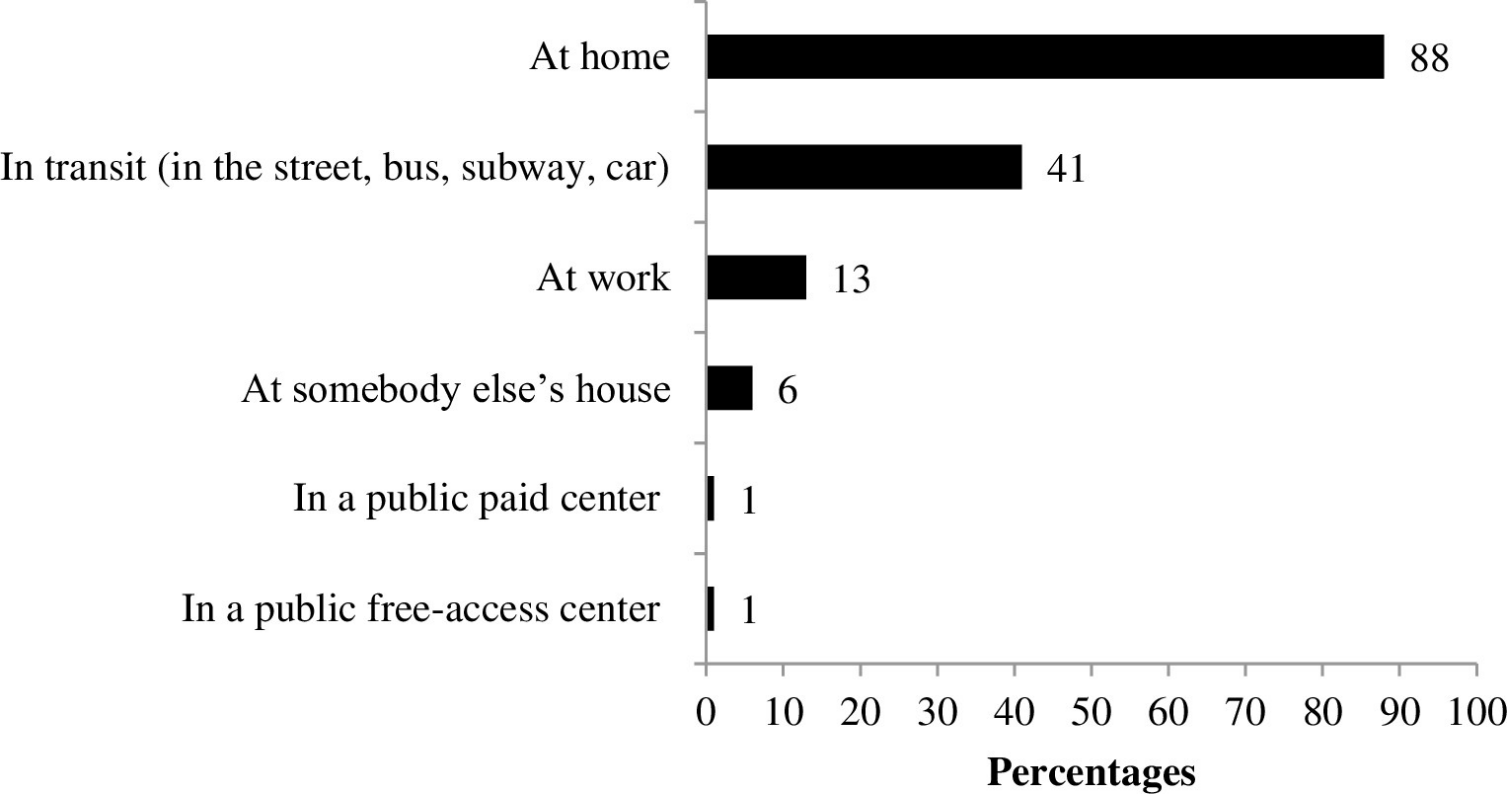
Proportion of Internet users by access location (% of total users, n = 101).

Among the participants, 91% (101/111) used the Internet, 57% (63/111) reported accessing it daily, 23% (26/111), at least once a week, and 4% (4/111), less than once a month. Only 9% (10/111) had never accessed the Internet. Among those who accessed the Internet (101/111), most did so from home (88%) (Fig 1). Cell phones were the most commonly used devices (72%), followed by notebooks (34%), desktops (32%) and tablets (13%).

When assessing the association between Internet use through smartphones and the variables (age, schooling and place of residence), it was observed that the proportion of users who used smartphones to access the Internet was high (over 60%), irrespective of place of residence or schooling, with no statistically significant difference between the different categories of these variables. The exception was the age variable, in which younger parents used smartphones to access the Internet significantly more than older ones (p < 0.05) (Table 2).

**Table 2:** Association between demographic data and mobile Internet use (n = 101).

Demographic data		Internet use on smartphone					p value
		Yes		No			
		n	%	n	%	Total (n)	
Place of residence	Greater São Paulo	44	(65)	24	(35)	68	0.061
	Interior of São Paulo	12	(86)	2	(14)	14	
	Other states	17	(89)	2	(11)	19	
Age group	24–34 years	35	(85)	6	(15)	41	0.003^a^
	35–44 years	28	(74)	10	(26)	38	
	45–59 years	10	(45)	12	(55)	22	
Schooling	Primary school	3	(60)	2	(40)	5	0.057
	High school	50	(68)	24	(32)	74	
	College	20	(91)	2	(9)	22	

^(a)^: Statistically significant difference; Fischer’s exact test (p < 0.05).

**Fig 2:**
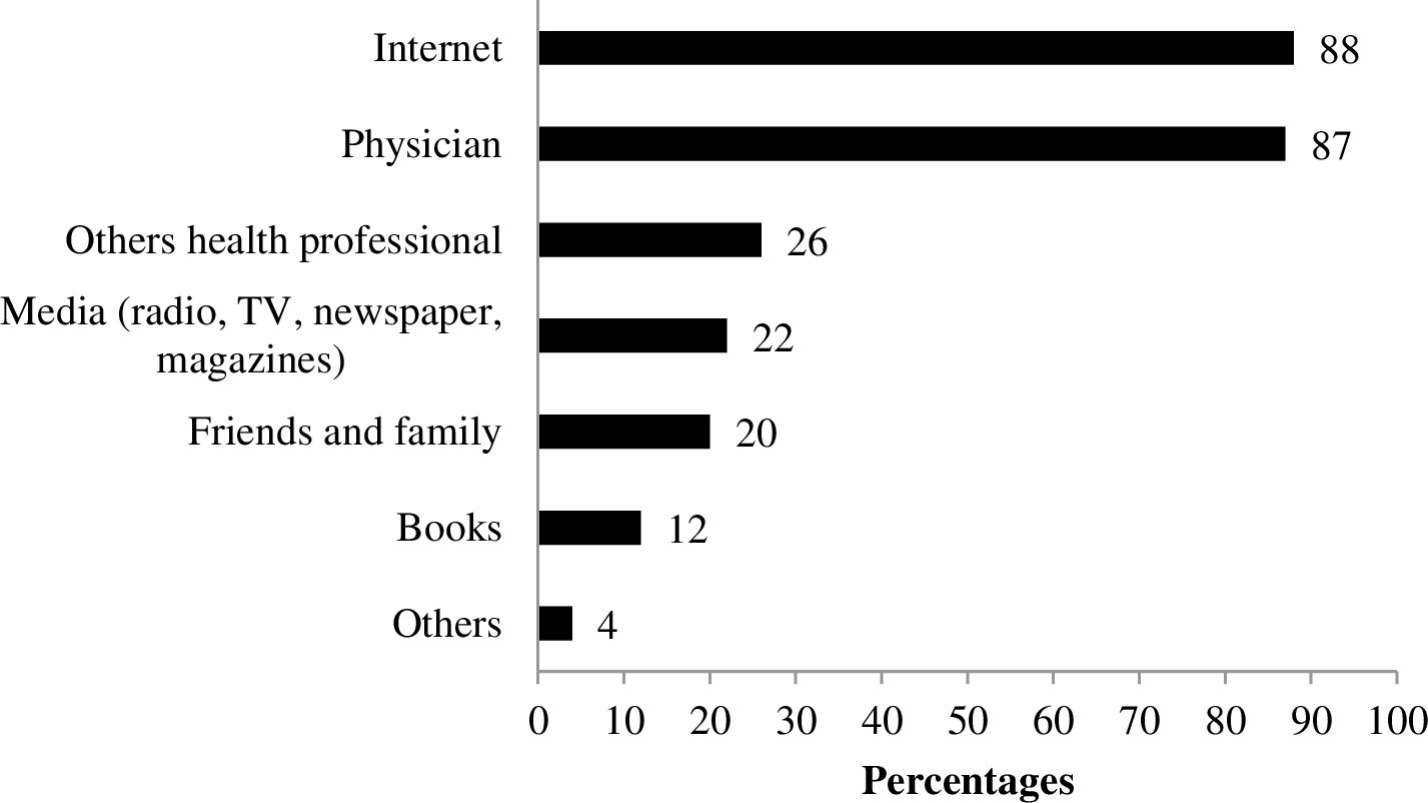
Sources of health information used by parents (%) (n = 101).

Of the 101 parents who reported using the Internet, 88% (89/101) said they used it to search for health information. Physicians continue to be the primary sources of information (87%, 88/101), but now they share space with the Internet (Fig 2).

Of the total number of parents who sought health information, 90% (80/89) used the Internet to search for information about CKD. The keyword most commonly used in the searches was “name of the disease” (86%, 69/80). As regards search results, 93% of these parents (74/80) considered the information useful, the majority of whom (78%, 58/74) believed that the information allowed a better understanding of the problem. Only 7% (6/80) did not find the information useful. The reasons listed were too much information, worry-generating information, and, lastly, excessively technical information. More than half (65%, 52/80) did not discuss with their physician that they had sought information on the Internet, 52% (27/52) of whom preferred not to discuss, 29% (15/52) claimed that the information was already covered by the physician, and 13% (7/52) forgot to discuss. For 85% (68/80), the information found was the same as that given by the physician (Table 3).

**Table 3:** Use profile and evaluation of the respondents regarding the health information found on the Internet.

		n	%
Have you used the Internet to access health information? (n = 101)	Yes	89	88%
	No	12	12%
Do you use the Internet to search for information about your child’s current health problem? (n = 89)	Yes	80	90%
	No	9	10%
Types of keywords used in the search (n = 80)	Name of the disease	69	86%
	Symptoms of the disease	8	10%
	Other keywords	3	4%
Did you find the information useful? (n = 80)	Yes	74	93%
	No	6	7%
Why was the information found useful? (n = 74)^(1)^	I got a better understanding of the problem	58	78%
	I confirmed the information I already had	25	34%
	I found support	7	9%
	I found the diagnosis	6	8%
Why was the information found not useful? (n = 6)^(1)^	Too much information	3	50%
	The information found made me worry	3	50%
	The information found was too technical	2	33%
Was the information found the same as that given by the doctor? (n = 80).	Yes	68	85%
	No	12	15%
Did you discuss with the doctor that you searched for information on the Internet? (n = 80)	Yes	28	35%
	No	52	65%
If you did not discuss, why? (n = 52)	I preferred not to discuss	27	52%
	The information was already covered by the doctor	15	29%
	I forgot to discuss	7	13%
	The information was not important	1	2%
	I did not have time	1	2%
	Other reason	1	2%

Note

(1) Parents could answer more than one option.

The level of interest of those seeking information about the disease was high, regardless of age, schooling, region of the country where they lived, or availability of mobile Internet access, with no statistically significant difference among the different categories of these variables (Table 4).

**Table 4:** Variables, categories and frequencies of responses regarding the use of the Internet by parents, to seek information about their child’s health problem (n = 89).

		Yes		No		p value
Variables		n	(%)	n	(%)	
Internet use on smartphone	No	19	(83)	4	(17)	0.229
	Yes	61	(92)	5	(8)	
Schooling	Primary school	2	(67)	1	(33)	0.269
	High school	57	(89)	7	(11)	
	College	21	(95)	1	(5)	
Age group	24–34 years	33	(89)	4	(11)	0.130
	35–44 years	31	(97)	1	(3)	
	45–59 years	16	(80)	4	(20)	
Place of residence	Greater São Paulo area	52	(88)	7	(12)	0.571
	Interior of São Paulo	14	(100)	0	(0)	
	Other states	14	(88)	2	(12)	

Fischer’s exact test (p < 0.05).

Among the reasons for performing the search, 94% (75/80) sought “for more information about the disease” and 53% (42/80) wanted to “know about the complications of the disease” (Table 5).

**Table 5:** Reasons to the use of the Internet to seek information about the child’s health problem (n = 80)^(1)^.

Reasons	%
To learn more information about the disease	94%
To learn about the complications of the disease	53%
To learn about the prognosis of the disease	48%
To learn about alternative treatments for the disease	16%
To seek medical treatment	14%
To seek patient support groups	14%
To clarify the information provided by the physician (“I didn’t understand”)	13%
To learn about possible diagnoses	13%
To seek other families having children with the same problem	10%
Not having enough time with the physician to make questions	10%
Being afraid to ask the physician	3%

Note

(1) Parents could answer more than one option.

## Discussion

To our knowledge, this is the first study on the Internet use pattern and influence of smartphone use for the search of health information by parents of children with CKD. The data show that almost all the parents interviewed had access to the Internet (91%) and used it daily (57%), or at least once a week (23%), to seek health (88%) and CKD information (90%).

Despite the fact that literature shows that parents of children with special needs seek more health information [15–20], the influence of the pattern of smartphone used for this purpose is still scarcely known [27]. This factor must be taken into account, especially in countries such as Brazil, where access to broadband Internet is not yet a reality for the entire population. The high cost of desktop computers, notebooks, and broadband Internet service in Brazil, limiting access by low-income individuals, has boosted the use of cell phones for this purpose (via mobile internet), and this use, in turn, has made possible the expansion of Internet access [14]. In our sample, the use of smartphones to access the Internet reached high percentages. Smartphones were the most commonly used devices to search information about health information and about CKD. The level of interest of those seeking information about the disease was high, regardless of availability of mobile Internet access, age, schooling and place of residence.

Since the sample of the present study was small and represents a population with specific characteristics, it was not possible to extrapolate to the Brazilian population as a whole. Interestingly, though, the percentage of Internet users seeking health information found in our survey (88%) was much higher than that found by the “ICT Households 2015” survey, according to which 41% of Internet users in Brazil sought information about health or health services [14].

Although in recent years, many people use the Internet to find medical information [12,13], the high percentage of search for information on CKD (90%) may be due to the fact reported by several authors [15–20] that parents of children with chronic, rare or severe problems seek information on the Internet more frequently. When it comes to CKD and its stages of severity, the need for information may be frequent. Another factor that may have contributed to the high search percentage is the fact that 90% of our sample was made up of mothers, considering that, according to Allen and Rainie [28], the main reason why mothers perform searches on the Internet is related to the health of their children.

As predicted by Cargill and Watson [3] in 2002, the Internet has become more important as a source of information about CKD. More than 15 years later, our data suggest that the Internet has not only surpassed traditional sources of information—such as health professionals other than physicians, the media (TV, radio, magazines), friends and family—but now it shares with the physician the status of the primary source of information preferred by parents.

Almost all the parents in our study sought more information about the disease and about its complications and prognosis, but only one-third reported having discussed about their initiative with the physician. Over the years, there seems to be some discomfort on the part of the patient’s parents in discussing with doctors that they had sought more information on the Internet, with steady percentages: 34% in 2002, in a study by Tuffrey and Finlay [23]; 43% in 2005, in a study by Boston et al. [29]; and 46.3% in 2015, in a study by Bao et al. [17]. The reasons given were mostly that parents preferred not to discuss with the physician or that the information was had already covered by the physician.

There is still room for improvement in communication between health professionals and parents, inasmuch as a significant—albeit relatively small (13%)—portion of parents reported not understanding the physician’s information and sought clarification using sources found on the Internet. On the other hand, we did not identify the main health information resources that can be found on the Internet. Almost all the parents (96%) of our sample said they knew the name of their child’s illness. But, since CKD may not be the only pathology afflicting the children and adolescents studied, it is not possible to establish whether the parents actually found the information they needed, when researching the name and symptoms of the disease, as reported. While it cannot be said that they always found the desired information, most felt that the information was useful, because it allowed a better understanding of the problem, confirming the observations of Ikemba et al. [15] and Sim et al. [24]. Only 7% did not consider the information useful, probably because they had difficulties to understand it, since they reported having found too much information or excessively technical information. It is possible that parents valued the information found as being useful, even if they did not find exactly what they were looking for. Although Zeng et al. [30] observed that the consumers of health information studied in their research were unable to find the desired information satisfactorily when performing a specific search, they nevertheless reported that these consumers regarded their Internet search experience as a positive one.

Given the importance of the Internet as a source of information, physicians and other health professionals must become aware of their role, understand it and use their knowledge and experience to assess the underlying evidence, the accuracy and the reliability of information available on the Internet, in order to guide parents and patients on how to access the best resources available on the web. According to Swallow et al. [6,7], parents of children with CKD emphasize the need for online support and information inasmuch as medical support is not always available. Better informed patients or parents can understand the disease and its treatment, perceive and report adverse effects, communicate problems and complaints, understand prescriptions and manage the disease more effectively. The widespread use of smartphones seen in our research should also be leveraged when developing web-based applications and resources to reach as many parents and patients as possible. The features of mobility, portability and interactivity render the smartphone a key-resource for the massive dissemination of health knowledge and education.

The limitations of our study are related to the research design. Firstly, inasmuch as it was a retrospective study, it is subject to memory bias. Secondly, although a significant part of the participants (33%) came from the most diverse regions of Brazil, inclusion of individuals from a single service made it difficult to generalize the results for all parents of patients with CKD. On the other hand, to the best of our knowledge, this is the only study that investigated the pattern of health information search on the Internet, and the influence of smartphone use among the parents of children with CKD.

Future research: The fact that neither the content, nor the accuracy, nor even the credibility of the information available on the Internet was evaluated, further investigation is needed to assess the sources of information available to these parents and/or patients. A more thorough investigation with semi-structured interviews will allow a better understanding of the influence of health information search on behavior for decision making and disease management. Studies surveying children and adolescents with CKD may also make important contributions, in that not only parents, but also patients use electronic devices, especially smartphones, and access the Internet massively. Adolescents should also be involved in the process of seeking health information, especially those with CKD.

## Conclusions

This study showed that a significant proportion of parents of children with CKD have been using the Internet, largely through smartphones, to research the condition, irrespective of age, schooling, and place of residence. The Internet and mobile technology could be better explored by physicians and other health professionals to provide health information to the parents of patients, broadening their understanding of the disease and the importance of their role in managing and sharing care with the medical staff, in order to improve clinical outcomes.

## Supporting information

S1 Questionnaire Questionnaire applied to parents/caretakers in this study. (DOCX)
