# Supplementary material for: Patterns of Internet and smartphone use by parents of children with chronic kidney disease
Source: PLoS One. 2019 Feb 12;14(2):e0212163. doi: 10.1371/journal.pone.0212163 (PMC6372181; doi:10.1371/journal.pone.0212163)
Supplement: S1 Questionnaire — (DOCX) [file pone.0212163.s001.docx]

**S1 - Questionnaire**

**Section 1 – General Information**

**1. What is your relationship to the patient?**

[ ] father [ ] mother [ ] other caretaker (specify) ________________________________

**2.Which city do you live in?** ________________________________________________________

**3.What’s your age?**

[ ] <24 years old [ ] 25 to 34 years old [ ] 35 to 44 years old [ ] 45 to 59 years old [ ] 60 years old or more

**4. What is your level of education?**

[ ] Illiterate / preschool [ ] Primary School [ ] High School [ ] College

**Section 2 – The child´s health condition**

**5. Child’s age:** _______________________

**6. Do you know the name of your child´s health condition?**

[ ] Yes [ ] No

**Section 3 – Internet information**

**7. How often do you use the internet?**

[ ] daily

[ ] at least once a week

[ ] at least once a month

[ ] less than once a month

[ ] never accessed the Internet

**8. Do you have internet access****? (you can choose more than one option)**

[ ] at home

[ ] at work

[ ] at somebody else's house

[ ] in a public paid center (cybercafé, lan house or similar)

[ ] at school

[ ] in transit (in the street, bus, subway, car)

[ ] in a public free-access center (telecenter, library, community organization)

**9. Do you access the internet?** **(you can choose more than one option)**

[ ] desktop

[ ] notebook

[ ] tablet

[ ] cell phones

**10. Have you ever used the internet to access health information?**

[ ] Yes [ ] No

**11. What other information sources about health do you use? (you can choose more than one option)**

[ ] physician

[ ] friends and family

[ ] books

[ ] other health professional

[ ] media (radio, TV, newspaper, magazines)

[ ] others

**12.** **Do you use the Internet to search for information about your child’s current health problem?**

[ ] Yes [ ] No

**13. What website (search engine) did you use to search for health information?**

[ ] Google

[ ] Bing

[ ] Facebook

[ ] Twitter

[ ] Others

**14. What did you use as keywords for the search?**

[ ] Name of the disease

[ ] Symptoms of the disease

[ ] Others Keywords

**15. Why did you search for information on the internet? (you can choose more than one option)**

[ ] To learn more information about your child´s disease

[ ] To seek medical treatment

[ ] To clarify the information provided by the physician (“I didn’t understand”)

[ ] To learn about the prognosis of the disease

[ ] To learn about the complications of the disease

[ ] Not having enough time with the physician to make questions

[ ] Being afraid to ask the physician

[ ] To learn about possible diagnoses

[ ] To learn about alternative treatments for the disease

[ ] To seek patient support groups

[ ] To seek other families having children with the same problem

**16. Did you find the information useful?**

[ ] Yes [ ] No

**17. Why was the information found useful****? (you can choose more than one option)**

[ ] I found the diagnosis

[ ] I confirmed the information I already had

[ ] I got a better understanding of the problem

[ ] I found support

[ ] Others. Specify: _________________________________________________________

**18. Why was the information found not useful? (you can choose more than one option)**

[ ] To much information

[ ] I didn´t find the information

[ ] The information found made me worry

[ ] Too little information

[ ] The information found was too technical

**19. Was the information found the same as that was given by the doctor?**

[ ] Yes [ ] No

**20. Did you discuss with the doctor that you searched for information on the Internet?**

[ ] Yes [ ] No

**21. If you did not discuss it with your doctor, why?**

[ ] The information was already covered by the doctor

[ ] I forgot to discuss

[ ] I preferred not to discuss

[ ] The information was not important

[ ] I did not have time

[ ] Other reason
